# An unusual presentation of tyrosine hydroxylase deficiency

**DOI:** 10.1186/s40734-017-0065-z

**Published:** 2017-12-05

**Authors:** Linn E. Katus, Steven J. Frucht

**Affiliations:** Movement Disorders Division, Department of Neurology, Mount Sinai Hospital, 5 E 98th Street, 1st floor, New York, NY 10029 USA

**Keywords:** Tyrosine hydroxylase deficiency, Dystonia, Genetics

## Abstract

**Background:**

Dopa-responsive dystonia (DRD) has largely been associated with autosomal dominant mutations in the GCH1 gene leading to GTP cyclohydrolase 1 deficiency. More recently, a deficiency in tyrosine hydroxylase (TH) has been recognized to cause DRD. This is a rare disorder resulting from genetic mutations in the TH gene on chromosome 11. The phenotype ranges from DRD with complete resolution on levodopa to infantile parkinsonism and encephalopathy only partially responsive to levodopa. Here we discuss an adult with TH deficiency with a history of possible parkinsonism and dystonia responsive to levodopa, notable for a residual dynamic segmental dystonia.

**Case presentation:**

Our patient grew up in rural Myanmar with limited medical care. Childhood was normal except for episodic illness with difficulty moving and speaking. At 18 years he developed difficulty writing. At 21 years he could not speak, walk, or write and was taken to a city hospital. Multiple medications were tried without benefit until he received carbidopa/levodopa, to which he had a miraculous response. Since then he has attempted to come off medication, however after several weeks his symptoms returned. On presentation to us at 31 years he was taking 450 mg levodopa/day and 4 mg trihexyphenidyl/day. He had a dynamic dystonia in his neck and trunk, subtle at rest and prominent with walking. He exhibited a sensory trick when touching his hand to his chin; improvement occurred to a lesser degree when he imagined touching his chin, and to an even lesser degree when the examiner touched his chin. He had no parkinsonism. He underwent genetic testing which revealed a homozygous variant mutation in the TH gene (p.Thr494Met) leading to a diagnosis of autosomal recessive tyrosine hydroxylase deficiency.

**Conclusions:**

TH deficiency can cause a broad range of clinical symptoms and severity. As more cases are discovered, the phenotype expands. Here we describe a unique case of DRD and possible parkinsonism due to TH deficiency with residual symptoms of dystonia that was task dependent and responded to a sensory trick. In addition, while the history is limited, it is possible he may have had episodes similar to “lethargy-irritability crises” seen in more severe cases. In large part he fits within the milder form of TH hydroxylase deficiency.

**Electronic supplementary material:**

The online version of this article (10.1186/s40734-017-0065-z) contains supplementary material, which is available to authorized users.

## Background

Dopa-responsive dystonia (DRD) was first described by Segawa et al. in 1976, who reported a Japanese family with progressive dystonia and diurnal fluctuations that were responsive to levodopa [1]. Since then, the majority of DRD has been associated with an autosomal dominant mutation in the GCH1 gene on chromosome 14 leading to GTP cyclohydrolase 1 (GTPCH1) deficiency [2–5]. More recently, a deficiency in tyrosine hydroxylase (TH) has been recognized to cause DRD [6]. TH deficiency is a rare disorder resulting from genetic mutations in the TH gene on chromosome 11 [6, 7]. The phenotype for TH deficiency ranges from DRD with complete resolution of symptoms on levodopa to infantile parkinsonism and encephalopathy that is only partially responsive to levodopa [3, 7–9]. Here we present an adult with TH deficiency manifesting with generalized DRD with residual dynamic dystonia in the neck and trunk that was task-dependent and responded to a sensory trick.

## Case presentation

Our 31 year old male patient was born in a rural village in Myanmar with limited access to medical care. He recalls being frequently ill during his childhood, but otherwise had normal development. He had been diagnosed with malaria due to frequent flu-like symptoms. He also had episodes when he was unable to move properly. The duration of these episodes is unclear, likely weeks. He had one episode at four years old where he could not speak, followed by a full recovery. His childhood progressed with these episodes until the age of 18 years. At this time, he developed difficulty writing with his right hand and switched to writing with his left. Within a year he developed dysarthria, described as trouble pronouncing words and phonating, as if “only air came out.” He also had a mildly abnormal gait without tripping or falling. Up until this time he was taught by missionaries and learned how to read, write, and speak English, which he did well despite his symptoms. However, his symptoms progressed and by 21 years of age he was unable to talk, walk, or write. It is unclear if his symptoms were due to dystonia and/or rigidity and bradykinesia at this time, though both are possible. He was brought to the nearest city for hospitalization, during which time he was tried on multiple medications. It is unknown what was tried initially, except for trihexyphenidyl; he had no response to any of them. He was then started on carbidopa/levodopa with profound improvement in his motor and speech abilities. He was discharged without a clear diagnosis but continued on carbidopa/levodopa functioning near normal.

At the age of 24 years he fled Myanmar to Malaysia. Doctors evaluated him there and advised him to stop carbidopa/levodopa as his exam was normal. After about 1 month off the medication he started to develop difficulty walking and talking. He restarted carbidopa/levodopa and these symptoms improved. He tried to discontinue carbidopa/levodopa a few more times but with each attempt his symptoms would return within several weeks.

At the age of 29 years he moved to the United States and was diagnosed clinically with dopa-responsive dystonia. He had head imaging and blood work that were reportedly unremarkable. He was on 450 mg/day of levodopa at the time. His symptoms had no diurnal variation, were relatively consistent throughout the day, and only seemed to worsen when he was tired. Trihexyphenidyl 4 mg/day was added with little benefit.

He first presented to us in October 2016 at the age of 31 years. At this time he was on carbidopa/levodopa 10/100 mg 1.5 tablets three times per day and trihexyphenidyl 2 mg twice per day. On exam he had dystonia in his neck and trunk that was subtle at rest and became prominent with walking. He displayed posterior shift and flexion in the neck, and mild extension in the trunk. He exhibited a sensory trick with improvement in his posturing when touching his hand to his chin; this also occurred but to a lesser degree when he imagined touching his chin without actually touching it, and to an even lesser degree when the examiner touched his chin. He had mild improvement when walking backwards. While walking forward he also had increased arm swing with mild choreic movements in his fingers, right side greater than the left. The remainder of his general and neurological exam was normal, with no parkinsonism and no evidence of dystonia in his limbs at rest or with action (including writing) or in his face. His exam can be seen in the included video (additional files 1, 2, 3, 4, 5 and 6).
**Additional file 1: Voice.** This video was taken on October 3rd 2016. The patient was 31 years old and was taking 450mg levodopa/day and 4mg trihexyphenidyl per day. (MOV 18820 kb)

**Additional file 2: Writing.** At rest he has subtle dystonia in the neck with posterior shift and flexion. (MOV 15528 kb)

**Additional file 3: Walking.** While walking he has more pronounced posturing in the neck and some extension of the trunk. (MOV 18174 kb)

**Additional file 4: Sensory trick.** Sensory trick. He exhibits a sensory trick, touching his finger to his chin with improvement in posturing. This occurs to a lesser degree when he imagines the trick and even to a lesser degree when the examiner touches his chin. (MOV 19429 kb)

**Additional file 5: Walking backwards.** He has mild improvement when walking backwards. (MOV 19160 kb)

**Additional file 6: Imagined sensory trick.** We also note increased arm swing and mild choreic movements in his right more than left fingers. (MOV 12803 kb)


He was referred for genetic testing to further elucidate the etiology of his dystonia. A 16-gene dystonia panel was sent through Invitae, including ANO3 (DYT23), ATP1A3 (DYT12), GCH1 (DYT5A), GNAL (DYT25), PARK2 (AR juvenile parkinsonism), PNKD (DYT8), PRKRA (DYT16), PRRT2 (DYT10), SGCE (DYT11), SLC2A1 (DYT9 and 16), SLC6A3, SPR (DYT5B), TH (DYT5B), THAP1(DYT6), TOR1A (DYT1), and TUBB4A (DYT4). This revealed a homozygous missense mutation in the TH gene (p.Thr494Met, transcript NM_199292.2) leading to a diagnosis of autosomal recessive tyrosine hydroxylase deficiency. This mutation is likely pathogenic and has been previously reported in patients with more severe phenotypes [10, 11].

He has no known family history of neurological or medical disease, nor of consanguinity. He knew his mother, father, older sister, and three younger brothers well until the age of 21 years when he left his village for medical care. He has had little to no contact with them since.

## Discussion

### Clinical characteristics and response to treatment:

TH deficiency manifests a wide clinical spectrum and is due to autosomal recessive mutations in the TH gene on chromosome 11 [3, 6, 7]. It is rare with about 40 cases reported by 2010 and several more since then [3, 7, 9]. TH deficiency syndromes have been classified into various types. Furukawa and Kish described three groups: dopa-responsive dystonia (DRD), infantile parkinsonism with motor delay, and progressive infantile encephalopathy [3]. Willemsen et al. distinguished the syndromes as type A or type B, where type A is a “progressive hypokinetic-rigid syndrome with dystonia” and type B a “complex encephalopathy” [7]. There is overlap between these various classifications, with type A similar to the DRD form and type B similar to the more severe progressive infantile encephalopathy.

In TH deficiency DRD, symptom onset is between 1 and 7 years of age with normal development up to this point [3, 9]. Lower limb dystonia and difficulty walking are the most common presentations with gradual progression to generalized dystonia. They can also have bradykinesia, rigidity, and tremors (typically postural), and cognition is spared [3, 7, 9]. About one third of known TH deficiency DRD patients have diurnal fluctuations, with worsening of symptoms towards the end of the day and improvement in the mornings after sleep [3, 9]. Characteristically, TH deficiency DRD patients have a robust and complete response to levodopa and have not shown adverse motor side effects (such as motor fluctuations and dyskinesia) [3]. In addition, progression of disease is not evident with treatment [7]. The type A phenotype described by Willemsen et al. also includes infants who present with symmetric bradykinesia and rigidity and less dystonia [7]. When onset is prior to 1 year of age there may be some mild static mental retardation, while those with older onset have normal cognition [7].

The more severe forms – infantile parkinsonism with motor delay, progressive infantile encephalopathy, and type B complex encephalopathy – have a broader range of movement disorders as well as cognitive involvement. They also do not have as robust a response to levodopa as the mild forms and are hyper-sensitive to it, developing motor fluctuations and dyskinesia on low doses [3, 7, 9].

In infantile parkinsonism with motor delay, the pregnancy and early post-natal course are usually normal with clinical onset between three to 12 months [3]. These patients present with truncal hypotonia and parkinsonism; dystonia is usually present but to a lesser degree [3, 9]. They may also display upper motor signs with spasticity and hyper-reflexia [3, 9]. Oculogyric crises may occur and mental retardation is common [3]. Ptosis and mild autonomic symptoms may be present, and symptoms typically do not have diurnal fluctuations [3]. Levodopa can provide notable benefit, though not complete resolution of symptoms and it may take months to years for the maximum response to be realized [3]. Unlike the DRD or type A patients, these patients may developed severe dyskinesia at treatment onset limiting dose increases [3, 9].

Progressive infantile encephalopathy is clinically evident prior to 6 months of age [3, 12]. The majority of patients have fetal distress, feeding difficulty, hypotonia, and decreased growth [3, 7, 12]. With time, motor delays, truncal hypotonia and limb hypertonia, bradykinesia, hyper-reflexia, ptosis, and mental retardation become evident [3]. While baseline dystonia is not usually a significant feature, dystonic crises may occur every 4–5 days [3]. Excessive jerky movements, such as tremor and myoclonus, as well as oculogyric crisis have been described [7]. They may display “lethargy-irritability crises” with paroxysmal lethargy and autonomic symptoms alternating with irritability [3, 7, 12, 13]. Autonomic disturbances can manifest with diaphoresis, drooling, and instability of body temperature (particularly pyrexia) [7]. More so than other forms of TH deficiency, these patients are hyper-sensitive to levodopa and its use is limited by severe dyskinesia [3]. Diurnal fluctuations are not typical [3, 7].

There are three atypical forms of TH deficiency described in the literature thus far. In 2005 Diepold et al. described a female who developed parkinsonism, truncal hypotonia, dystonic hand posturing, and psychomotor developmental delay at 14 months of age after a viral illness [14]. She responded to levodopa, but with residual hypotonia and developmental delay. In 2007 Giovanniello et al. reported a case with a biphasic clinical course [15]. The male patient first developed toe walking, falls, and language delay at 2 years old, with prior normal development. Then at age 11 years he deteriorated with the emergence of involuntary choreic and myoclonic movements, oculogyric crises, dysarthria and dysphonia, slow speech, a masked face, eye movement abnormalities, and cognitive impairment. He responded to levodopa but the dose was limited by side effects. A third atypical presentation was published in 2012 by Stamelou et al. describing three siblings with a myoclonus-dystonia syndrome [16]. They presented with hypotonia around 6 months old and progressed to develop severe myoclonus, dystonia, and oculogyric crises. They responded in part to levodopa and were unique genetically in that they had compound heterozygosity with one previously known mutation in the promoter region and another novel nonsynonymous mutation in the other allele of the TH gene.

Our patient’s clinical presentation most closely fits with DRD or the type A phenotype, however he has a few unique features. Though the history is limited, he describes episodes of feeling ill with difficulty walking and speaking lasting days to weeks, as well as other periods of flu-like illness. He had a diagnosis of malaria and it is unclear if symptoms were due to this or his TH deficiency; if the latter, these episodes may be akin to the lethargy-irritability crises or dystonic crises previously described in the literature. Once he was treated with levodopa at the age of 21 years he showed profound response, allowing him to function normally. However, he has never had a complete response, remaining symptomatic with segmental dystonia in his neck and trunk without diurnal variation. This dystonia is task-dependent, most prevalent while walking; it improves with a sensory trick and to a lesser degree with walking backwards.

### Work-up and diagnosis:

The differential diagnosis for DRD includes GTPCH1 deficiency and sepiapterin reductase (SR) deficiency, in addition to TH deficiency. GTPCH1 deficiency, or Segawa syndrome, is a more common and well-known cause of DRD. It is due to an autosomal dominant mutation in the GCH1 gene, leading to a deficiency of GTPCH1 [3, 10]. SR deficiency, on the other hand, is rare and autosomal recessive. It is due to a mutation in the SPR gene on chromosome 2 resulting in a deficiency of SR [17]. SR deficiency typically presents in infancy and phenotypically may be similar to the severe forms of TH deficiency [17, 18].

Cerebrospinal fluid (CSF) analysis can help distinguish these monoamine neurotransmitter syndromes. TH catalyzes the conversion of tyrosine to levodopa, which is then converted to dopamine by aromatic amino acid decarboxylase (AADC). Dopamine is broken down into homovanillic acid (HVA) and is converted into norepinephrine and epinephrine. These two catecholamines are broken down into 3-methoxy-4-hydroxyphenylethyline glycol (MHPG). With decreased TH activity, there is less levodopa, leading to less dopamine, norepinephrine, and epinephrine, and ultimately decreased levels of HVA and MHPG, which can be detected in the CSF. The tryptophan to serotonin to 5-hydroxyindolecacetic acid (5-HIAA) pathway, however, is unaffected in TH deficiency. Thus, 5-HIAA levels in the CSF are normal. CSF levels showing decreased HVA and MHPH with normal 5-HIAA, and thus a low HVA/5-HIAA ratio, are highly suggestive of TH deficiency. These levels also correlate with clinical severity of disease [7]. We did not test our patient’s CSF as he was sent for definitive genetic testing instead.

GTPCH1 and SR are involved in the synthesis of BH4, which is essential to the activity of TH and phenylalanine hydroxylase in dopamine synthesis and tryptophan hydroxylase in serotonin synthesis [2, 3, 19]. Thus, low levels of both HVA and 5-HIAA, as well as BH4, can be seen [2, 20]. GTPCH1 deficiency is distinguished from SR deficiency by CSF levels of biopterin and neopterin: they are low in GTPCH1 deficiency, but are high and normal (respectively) in SR deficiency [2, 10, 20, 21].

Neuroimaging in TH deficient patients is generally normal [3, 22]. Those with infantile encephalopathy may have nonspecific diffuse atrophy or periventricular white matter changes on brain MRI [3, 13].

The definitive diagnosis can be made through genetic testing, revealing autosomal recessive mutations in the TH gene on chromosome 11 [3, 7]. These can be either homozygous or compound heterozygous, which lead to decreased TH function [3]. A complete block of function would result in perinatal death [3]. There are more than 50 known pathogenic mutations [3, 10].

## Conclusions

TH deficiency can cause a broad range of clinical symptoms and severity. As more cases are discovered, the phenotype expands. Here we describe a unique case of DRD and possible history of parkinsonism caused by TH deficiency due to a homozygous missense mutation in the TH gene (p.Thr494Met). In large part, phenotypically he fits within the milder form of TH hydroxylase deficiency, save a few discrepancies. His disorder does not show complete response to levodopa and his residual symptoms behave like an idiopathic segmental dystonia that is task-dependent and responds to a sensory trick. In addition, while the history is limited, it is possible he may have had episodes similar to “lethargy-irritability crises” or dystonic crises seen in more severe cases. His genetic mutation has been described in the past, though associated with a more severe phenotype [10, 11]. As more cases are discovered it is important to clearly identify distinguishing features of TH deficiency to allow for proper diagnosis and treatment.

## Abbreviations

5-HIAA: 5-hydroxyindolecacetic acid
AADC: aromatic amino acid decarboxylase
BH4: tetrahydrobiopterin
CSF: cerebrospinal fluid
DRD: dopa-responsive dystonia
GTPCH1: GTP cyclohydrolase 1 deficiency
HVA: homovanillic acid
MHPG: 3-methoxy-4-hydroxyphenylethyline glycol
TH: tyrosine hydroxylase deficiency
